# Kidney and Urinary Tract Involvement in Epidermolysis Bullosa: Is Routine Follow-Up Necessary?

**DOI:** 10.5826/dpc.1103a51

**Published:** 2021-05-20

**Authors:** Neslihan Cicek, Nurdan Yildiz, Ruslan Asadov, Ayse Deniz Yucelten, Halil Tugtepe, Harika Alpay

**Affiliations:** 1 Department of Pediatric Nephrology, Marmara University School of Medicine, Istanbul, Turkey; 2 Department of Radiology, Marmara University School of Medicine, Istanbul, Turkey; 3 Department of Dermatology, Marmara University School of Medicine, Istanbul, Turkey; 4 Department of Pediatric Urology, Marmara University School of Medicine, Istanbul, Turkey

**Keywords:** end stage renal disease, epidermolysis bullosa, peritoneal dialysis, urinary tract involvement

## Abstract

**Background:**

Several renal and urinary tract complications have been reported in patients with epidermolysis bullosa.

**Objective:**

This study investigated kidney and urinary tract involvement in patients with epidermolysis bullosa.

**Patients and Methods:**

Patients with epidermolysis bullosa in treatment at the Dermatology Unit were included in the study. Glomerular and tubular functions were investigated.

**Results:**

The study included 16 patients (4 females, 12 males) of mean 11.1 years (SD = 8.1 years). Estimated GFR was normal in all patients except one with end-stage renal disease. Excluding this patient, the urinary albumin/creatinine ratio and the fractional excretion of sodium were normal. The mean beta-2 microglobulin/creatinine ratio was 278.8 μg/g, and it was abnormally high in 2 patients. The mean tubular phosphorus reabsorption was 92.6%; it was abnormally low in 1 patient. Severe kidney or urinary tract involvement was present in 2 patients with recessive dystrophic EB-generalized severe (RDEB-GS): one patient had obstructive bullous lesions in the urethra; the other had end-stage renal disease secondary to focal segmental glomerulosclerosis and was on peritoneal dialysis for 3 years.

**Conclusions:**

Assessment for renal and urinary tract involvement should become a routine part of the evaluation of patients with any type of EB, but especially of patients with RDEB-GS. Patients with mild tubular dysfunction need long-term follow-up to detect early deterioration of renal function.

## Introduction

Epidermolysis bullosa (EB) is a rare inherited disorder characterized by fragility to minor trauma, resulting in bullous skin lesions that may be associated with variable systemic involvement. Currently, EB is classified into simplex, junctional, dystrophic types and Kindler syndrome (mixed cleavage plane) according to the ultrastructural cleavage plane in skin and location of the target protein [1,2]. Currently, immunofluorescence antigen mapping, transmission electron microscopy and genetic analysis are recommended to confirm the diagnosis as well as to classify the type and subtype of EB [2,3]. These techniques are largely inaccessible in developing countries. Therefore, Yenamandra et al [4] developed a clinical diagnostic matrix that indicates the presence or absence of different features for the diagnosis of 9 EB subtypes [4].

In patients with EB, respiratory, genitourinary and gastrointestinal system involvement has been described in isolated case reports and small case series. Several renal complications, including IgA nephropathy, amyloidosis, post-infectious glomerulonephritis, hereditary nephritis, and upper and lower urinary tract obstructions, have been reported [5–10]. These complications may lead to chronic kidney disease with high morbidity and mortality [6–8]. Although renal and urinary tract involvement is usually seen in patients with the most severe subtypes (eg junctional and recessive dystrophic disease), these complications may arise in any subtype of inherited EB, including the milder forms [7,11].

Patients with EB mostly refer to dermatology and pediatric outpatient clinics and are mainly concerned with skin and related complications. Therefore, awareness of pediatricians about nephrourological involvement of EB is important for the early diagnosis and treatment of complications. In this study, we investigated renal and urinary tract involvement in a series of patients with EB.

## Materials and Methods

Sixteen patients with EB, followed at the Dermatology Unit, were involved in the study. Two patients were referred to the Pediatric Nephrology Unit, and the other 14 patients had no history of kidney or urinary tract involvement. The study was approved by the Ethics Committee of Marmara University School of Medicine (09.2016.242) and was done in accordance with the Declaration of Helsinki. After a full explanation of the study was given to parents and children, informed consent was obtained from the parents for children and from patients for adults.

Demographic data were recorded from the medical files of the patients. As electron microscopy, immunologic studies and genetic analyses were not available due to technical and financial difficulties, the clinical diagnostic matrix of Yenamandra et al [4] was used to diagnose the EB subtype. Distribution of lesions, excessive granulation tissue, scarring, milia, nail involvement, mucosal involvement, poor dental enamel, keratoderma, chronic wounds, syndactyly, alopecia, poikiloderma, relative growth failure, survival after 2 years and affected parents were evaluated in this clinical diagnostic matrix.

In all patients, fasting morning blood samples and spot urine samples were obtained. Serum creatinine, cystatin C, sodium, phosphorus, 24-hour urinary sodium, phosphorus, creatinine, albumin and beta-2 microglobulin (B2M) were measured. Glomerular functions were evaluated from serum creatinine, cystatin C, estimated glomerular filtration rate (eGFR) and urinary albumin/creatinine ratio. eGFR (normal, ≥ 90 mL/min 1.73 m^2^) was calculated from height and plasma creatinine using a modified Schwartz formula (0.413 × height (cm)/plasma creatinine (mg/dL) [12].

Renal tubular functions were assessed by determination of fractional excretion of sodium (FENa), tubular phosphorus reabsorption (TRP) and the urinary B2M/creatinine ratio. FENa (normal, <1 %) was calculated with the formula (urinary sodium (mmol/L) × plasma creatinine (mg/dL))/(plasma sodium (mmol/L) × urinary creatinine (mg/dL)). TRP (normal, ≥85 %) was calculated with the formula 1 - (urinary phosphorus (mg/dL) × plasma creatinine (mg/dL))/(plasma phosphorus (mg/dL) × urinary creatinine (mg/dL)). The urinary B2M/creatinine ratio was considered normal when <300 μg/g, and the urinary albumin/creatinine ratio was normal when <30 mg/g. Urinary system ultrasonography was performed in all patients by the same radiologist.

## Results

The study evaluated 16 EB patients (12 males and 4 females) of mean age 11.1 years (SD = 8.1 years; range, 1.6–28.7 years). The most common types of EB were recessive dystrophic EB–generalized severe (RDEB-GS, 7 cases), junctional EB–generalized severe (JEB-GS, 3 cases) and EB simplex–localized (EBS-L, 2 cases) (Table 1).

Kidney or urinary tract involvement was observed in 4 patients, including 1 case (#11) with focal segmental glomerulosclerosis and end-stage renal disease (ESRD), 1 case (#1) with urethral obstruction and hydroureteronephrosis, and 2 patients (#5 and #12) with mild renal tubular dysfunction.

The patient with ESRD (#11) was excluded from further analysis. For the remaining 15 cases, the mean values of serum creatinine, cystatin C and eGFR were 0.38 mg/dL (SD = 0.15), 0.7 mg/dL (SD = 0.09) and 182 mL/min 1.73 m^2^ (SD = 52.4), respectively, and were considered normal. The mean albumin/creatinine ratio was 15.3 mg/g (SD = 17.2), and was normal in the 15 patients. The mean urinary B2M/creatinine ratio, an indicator of renal tubular function, was 278.8 μg/g (SD = 577); it was high in 1 patient with EB simplex–generalized severe (EBS-GS, #5) and 1 patient with RDEB-GS (#12). FENa was less than 1% and normal in all patients (excluding patient #11). The mean TRP was 92.6% (SD = 3.8%); it was slightly low in a patient with RDEB-GS (#2). Overall, 13 patients had normal ultrasonography findings.

The patient who had urinary tract involvement was a 17.7 year-old boy with RDEB-GS (#1). He was admitted to hospital with dysuria and a poor urinary stream. Ultrasonography revealed bilateral grade 1 hydroureteronephrosis. The skin lesions were present since birth and he had no previous urinary tract infection. Urine cultures were sterile, and glomerular and tubular function tests were normal (Table 1). Uroflowmetry revealed a serious obstructive pattern, with a maximum flow rate of 4 mL/s, a voided volume of 75 mL and a voiding time of 471 s. Cystoscopy was performed, and several bullous lesions in the bulbar and prostatic urethra, causing partial obstruction, were resected. After the procedure, his symptoms persisted and he was unable to empty his bladder completely. Therefore, clean intermittent catheterization was suggested due to a high post-voiding residual urine volume. He couldn’t tolerate urethral catheterization, so underwent a Monti procedure (creation of a conduit between the bladder and skin using part of the gastrointestinal tract). He is still on clean intermittent catheterization by Monti procedure.

**Figure 1:**
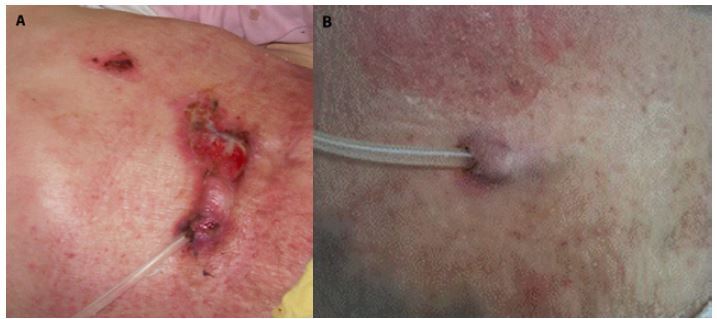
Peritoneal catheter exit site in patient #11, (A) on day 10 and (B) after 2 months

The patient with RDEB-GS (#11) and renal involvement was a 20-year-old female with digital fusion, lower limb contractures and impaired mobility. She was referred to our pediatric nephrology clinic because of high serum creatinine (1.4 mg/dL) and proteinuria (14.3 mg/m^2^ h) at the age of 16 years. Renal ultrasound demonstrated kidneys with increased echogenicity. She underwent renal biopsy without complications, and histopathological examination revealed focal segmental glomerulosclerosis with 30% global sclerosis. Amyloidosis and immunofluorescence microscopy were negative. Her renal functions deteriorated progressively and ESRD developed (serum blood urea nitrogen, 70 mg/dL; serum creatinine, 6.97 mg/dL; and eGFR = 10.2 mL/min 1.73 m^2^). Peritoneal dialysis was preferred to avoid recurrent vein punctures. A double cuffed swanneck Tenckhoff catheter was inserted into the peritoneal space and continuous ambulatory peritoneal dialysis was started. Although she had recurrent blistering of most parts of her body, the exit site was clean (Figure 1). She had only one attack of peritonitis with sterile culture during follow-up. She was not able to perform dialysis herself because of severe syndactyly, and therefore her mother performed the dialysis exchanges.

The patient with Kindler syndrome (#16) was a 7-year-old boy. Ultrasonography revealed a small left kidney. Radionuclide scanning with dimercaptosuccinic acid demonstrated a split function of 18% on the left kidney without scar tissue. He was diagnosed as having a hypoplastic left kidney.

## Discussion

EB is a heterogeneous, hereditary skin disorder with severe cutaneous and extracutaneous involvement. Mucosal lesions can be observed in the mouth, larynx, esophagus, bronchia, large bowel, urogenital tract, vagina and anus [1,2]. There are only a few case reports and small case series regarding kidney and urinary tract involvement in EB. Kidney and urinary tract involvement has been described in all EB types, particularly in dystrophic EB and junctional EB [13]. In our study, we evaluated renal functions and urinary tract involvement of 16 patients with EB.

Nephropathy is a serious complication of the major types of EB, particularly severe RDEB. The kidneys may be injured either by obstruction due to mucosal lesions or by glomerular disease [8,10]. In our series, one patient with RDEB-GS had bullous lesions within the bulbar and prostatic urethra, causing partial obstruction of outflow. It is reported that genitourinary involvement may vary from meatal stenosis leading to upper tract dilatation to severe stenosis due to bullous lesions and scarring at the ureterovesical junction requiring permanent urinary diversion [14,15]. The mechanisms may include chronic blister formation within the lining epithelia of the urinary tract, and chronic systemic inflammation [16]. The most common type in these cases is either junctional or dystrophic EB, which have serious multisystem involvement. Fine et al reported that urinary retention and hydronephrosis occurred in 9.3 % and 7 % of cases, respectively, of junctional EB [7]. Obstructive lesions may lead to chronic renal injury if left untreated.

Surgery for urethral lesions in EB patients has not been clearly described. Chan et al reported a patient with obstructive uropathy and described surgical treatments in detail [10]. In our study, obstructive bullous lesions within the bulbar and prostatic urethra were resected from one young male patient. However, his symptoms did not regress, and catheterization was suggested due to the high post-voiding residual urine volume. It is important to note that the use of instruments in the urinary tract might cause serious lesions, including stenosis, and may worsen the clinical course, so should be avoided as much as possible [9]. The clinical course of this patient worsened; because he could not tolerate urethral catheterization, he underwent a Monti procedure (creation of a conduit between the bladder and skin).

With a significant increase in survival of EB patients, renal complications are seen more frequently. There are a few publications, mostly case reports, on kidney involvement in EB (Table 2). Chronic kidney disease has most commonly been reported in patients with RDEB and junctional EB. Fine et al reported the mortality of patients with junctional EB and RDEB due to ESRD as 0.52 % and 1.4 8%, respectively [8]. Chronic post-infectious glomerulonephritis attributed to recurrent superinfections of bullous skin lesions, mesangioproliferative glomerulonephritis, IgA nephropathy, and nephrotic syndrome secondary to amyloidosis leading to ESRD have all been reported in EB patients [5,10,15,16,17,18]. Although we have a limited number of cases, one of our patients (patient #11 with RDEB-GS) had ESRD secondary to focal segmental glomerulosclerosis. Even though invasive procedures should be avoided in these patients, renal biopsy may be performed successfully if necessary. Focal segmental glomerulosclerosis is rare in patients with EB [19].

The mechanism of glomerular involvement is still not fully understood. Compositional changes of various proteins involved in the development, structure, and function of both skin and kidney (eg integrins, laminin, collagen XVII, CD151) alter glomerular cell-matrix interactions and may result in increased glomerular capillary wall permeability through abnormal podocyte adhesion to the glomerular basement membrane or alteration in the structure of the filtration slit diaphragms [13,16,20,21]. Mutations in the gene for integrin α3 (*ITGA3*), the main integrin linking podocyte foot processes to the glomerular basement membrane, cause junctional EB with interstitial lung disease and renal anomalies (ILNEB, OMIM#614748) including congenital nephrotic syndrome, focal segmental glomerulosclerosis, bilateral renal cysts, and a spectrum of congenital anomalies of the kidney and urinary tract (CAKUT) [13,21]. Focal segmental glomerulosclerosis and CAKUT were described in junctional EB with *ITGB6* and *ITGB4* mutations leading to abnormal integrin β6 and β4 subunits [19,21].

Mutations in genes encoding type XVII collagen (*COL17A1*) and laminin 332 (*LAMA3, LAMB3* and *LAMC2*), which are expressed in renal glomerular podocytes and bind to integrins, were reported in patients with junctional EB and renal involvement [20–22]. Type VII collagen interacts with laminins and integrins in the kidney. Chronic renal failure, post-streptococcal glomerulonephritis, IgA mesangial disease and renal amyloidosis have been reported in patients with the recessive forms of dystrophic EB, with *COL7A1* mutations resulting in markedly reduced or abnormal type VII collagen [13,16]. CD151, a ligand of integrins expressed in glomerular podocytes, contributes to cell adhesion and maintenance of the glomerular basement membrane. Homozygous mutations in *CD151* gene encoding tetraspanin CD151 were reported in patients with EB simplex and nephropathy [20,21,23]. It is believed that chronic inflammation causes glomerulonephritis due to immune complex formation or extensive immunoglobulin synthesis [24].

In our patients (excluding case #11), the mean eGFR was high (182 mL/min 1.73 m^2^). This finding seems to be due to hyperfiltration.

To our knowledge, renal tubular functions in EB have not been fully evaluated. The etiopathogenesis of tubular dysfunction may include frequent nephrotoxic antibiotic therapies to treat skin infections and chronic inflammation. Hata et al [22] reported high urinary albumin, *N*-acetylglucosaminidase and B2M excretion, indicating glomerular and renal tubular involvement, in a patient with Herlitz junctional EB and a *LAMB3* gene mutation. They showed the absence of the α3 subunit of laminin-5 in tubular basal membranes, and suggested that the lack of laminin-5 in the renal tubular basement membrane was responsible for renal tubular involvement [22]. We found mild tubular dysfunction in 2 patients with normal eGFR. Although our study group was small, these data emphasize the importance of monitoring tubular functions by testing for low molecular weight proteinuria and by calculating FENa and TRP, to detect early deterioration of kidney functions and prevent end-organ damage, even though the majority of urinary tract involvement in EB patients may be asymptomatic before renal dysfunction develops. However, our results need to be expanded upon in large multicenter studies.

The choice of hemodialysis or peritoneal dialysis for EB patients with ESRD should be decided on an individual basis, taking into consideration patient and familial performance status and available facilities. Peritonitis, intraperitoneal adhesions, exit site complications and sepsis limit the use of peritoneal dialysis in many patients. In this study, one patient (#11) was treated with peritoneal dialysis for 3 years without serious complications. Chan et al [10] reported 2 RDEB patients treated with peritoneal dialysis: in the first case, peritoneal dialysis had to be changed to hemodialysis due to peritoneal infections and adhesions; the second patient died from sepsis 4 months after catheterization. Ahmadi and Antaya [25] reported a successful 14-month period of peritoneal dialysis without peritonitis or exit site complication in a 29-year-old woman with RDEB-GS and IgA nephropathy. We preferred peritoneal dialysis in our patient to avoid recurrent vein punctures because of her skin lesions. At the beginning, we were concerned about her catheter exit site; however, she tolerated dialysis well, the exit site improved, and no exit site complications such as infection, blisters or bleeding were observed (Figure 1).

In this study, we observed 2 important complications in patients with RDEB-GS. Although our study has a limited number of patients, we suggest that patients with any type of EB may have renal and urological complications that may lead to chronic renal injury.

A major limitation of our study is that diagnoses of the patients were not confirmed by immunofluorescence, electron microscopic and genetic studies due to technical and financial difficulties and health care system limitations. Instead, we used a clinical diagnostic matrix [4]. On the other hand, kidney involvement and urinary tract complications may arise in any subtype of EB, including milder forms [7,11,13]. Therefore, the clinical diagnosis does not change the message that we emphasize in this paper. We aim to draw attention to kidney and urological problems with this study, since EB patients are mostly followed-up in dermatology and pediatric outpatient clinics and are mainly concerned with skin involvement.

## Conclusions

Considering the significant increase in survival and longevity of EB patients, we strongly recommend that evaluation of glomerular and tubular functions and assessment of possible kidney-urinary tract involvement become a routine part of the diagnostic work-up of EB patients. Milder complications may arise in any subtype of inherited EB. Children with mild tubular dysfunction need long-term follow-up to detect early deterioration of kidney functions and to prevent organ damage later in life.

## Figures and Tables

**Table 1:** Clinical Characteristics of 16 Patients with Epidermolysis Bullosa

Case	Sex	Diagnosis^1^	Age (years)	eGFR^2^	Urinary albumin/creatinine ratio	Urinary B2M/creatinine ratio	TRP	(%) FENa (%)
1	M	RDEB-GS	17.7	135	7.7	77	92	0.88
2	M	RDEB-GS	11	101	11.2	40	82	0.3
3	M	EBS-L	4.7	189	11.1	50	97	0.25
4	M	RDEB-GI	14.5	286	7.1	83	93	0.04
5	M	EBS-GS	20.2	136	16.5	390	93	0.63
6	F	EBS-L	28.7	172	6.78	49.2	90	0.04
7	M	RDEB-GS	14.2	191	9.6	98	91	0.43
8	M	EBS-GI	4.5	205	75	102	95	0.26
9	M	JEB-GS	4.5	250	11	302	90	0.07
10	M	RDEB-GS	6	180	8.2	250	92	0.02
11^3^	F	RDEB-GS	20.7	3.7	1	5000	32	25.6
12	F	RDEB-GS	22	265	9.1	2332	92.5	0.75
13	M	JEB-GS	1.6	198	25.3	65.8	98	0.17
14	M	JEB-GS	7	150	10.9	41	95	0.33
15	F	RDEB-GS	2.5	148	15.3	150	93	0.56
16	M	Kindler syndrome	7.2	141	5.46	153	96	0.61

EB = epidermolysis bullosa; EBS-GI = EB simplex-generalized intermediate; EBS-GS = EB simplex-generalized severe; EBS-L = EB simplex-localized; eGFR = estimated glomerular filtration rate; F = female; FENa = fractional excretion of sodium; JEB-GS = junctional EB-generalized severe; M = male; RDEB-GI = recessive dystrophic EB-generalized intermediate; RDEB-GS = recessive dystrophic EB-generalized severe; TRP = tubular phosphorus reabsorption.

1 Based on clinical findings and the clinical diagnostic matrix [11].

2 Expressed in units of mL/min 1.73 m^2^. Normal values are ≥90.

3 This patient had end-stage renal disease.

**Table 2:** Reported Cases of Renal Involvement in Patients with Epidermolysis Bullosa

Sex	Age (years)	EB type	Renal involvement	Dialysis	Outcome	Ref.
M	14	RDEB-HS	IgA nephropathy	Hemodialysis	On follow-up	[5]
M	14.5	RDEB	MPGN	Peritoneal	Exitus	[10]
F	11	RDEB	IgA nephropathy	Hemodialysis and peritoneal	Exitus	[10]
M	20	RDEB	IgA nephropathy	No	Exitus	[15]
F	29	RDEB	IgA nephropathy	Peritoneal	On follow-up	[25]
M	19	RDEB	Amyloidosis	NA	NA	[26]
M	22	RDEB	Amyloidosis	NA	NA	[26]
F	27	RDEB	Amyloidosis	NA	NA	[26]
F	30	RDEB	Amyloidosis	NA	NA	[26]
F	17	DEB	IgA nephropathy	No	On follow-up	[27]
M	10	DEB	PIGN	No	On follow-up	[28]
F	17	DEB	Amyloidosis		Exitus	[28]
F	15	RDEB	Nephrotic syndrome, amyloidosis	No	On follow-up	[29]
M	38	RDEB	Amyloidosis	Hemodialysis	NA	[30]
M	4	NA	IgA nephropathy	No	On follow-up	[31]
M	33	JEB	IgA nephropathy	Hemodialysis	Transplantation	[32]
M	27	RDEB	IgA nephropathy	No	Transplantation	[33]
M	6	RDEB	Nephrotic syndrome, amyloidosis	No	On follow-up	[34]
M	39	DEB	MPGN	No	On follow-up	[35]

DEB = dystrophic epidermolysis bullosa; EB = epidermolysis bullosa; F = female; HS = Hallopeau-Siemens; JEB = junctional epidermolysis bullosa; M = male; MPGN = membranoproliferative glomerulonephritis; NA = not available; PIGN = post-infectious glomerulonephritis; RDEB = recessive dystrophic epidermolysis bullosa.
